# Incremental Versus Immediate Induction of Hypertension in the Treatment of Delayed Cerebral Ischemia After Subarachnoid Hemorrhage

**DOI:** 10.1007/s12028-022-01466-7

**Published:** 2022-03-08

**Authors:** Michael Veldeman, Miriam Weiss, Walid Albanna, Omid Nikoubashman, Henna Schulze-Steinen, Hans Clusmann, Anke Hoellig, Gerrit Alexander Schubert

**Affiliations:** 1 Department of Neurosurgery, RWTH Aachen University, Aachen, Germany; 2 Department of Diagnostic and Interventional Neuroradiology, RWTH Aachen University, Aachen, Germany; 3 Department of Intensive Care Medicine, RWTH Aachen University, Aachen, Germany; 4 Department of Neurosurgery, Kantonsspital Aarau, Aarau, Switzerland

**Keywords:** Subarachnoid hemorrhage, Induced hypertension, Delayed cerebral ischemia, Delayed cerebral infarction

## Abstract

**Background:**

Delayed cerebral ischemia (DCI) is a common complication of aneurysmal subarachnoid hemorrhage and contributes to unfavorable outcome. In patients with deterioration despite prophylactic nimodipine treatment, induced hypertension (iHTN) can be considered, although the safety and efficacy of induction are still a matter of debate. In this study, two iHTN treatment algorithms were compared with different approaches toward setting pressure targets.

**Methods:**

In a cohort of 325 consecutive patients with subarachnoid hemorrhage, 139 patients were treated by induced hypertension as a first tier treatment. On diagnosing DCI, blood pressure was raised via norepinephrine infusion in 20-mm Hg increments in 37 patients (iHTN_incr_), whereas 102 patients were treated by immediate elevation to systolic pressure above 180 mm Hg (iHTN_imm_). Treatment choice was based on personal preference of the treating physician but with a gradual shift away from incremental elevation. Both groups were evaluated for DCI-caused infarction, the need of additional endovascular rescue treatment, the occurrence of pressor-treatment-related complications, and clinical outcome assessed by the extended Glasgow outcome scale after 12 months.

**Results:**

The rate of refractory DCI requiring additional rescue therapy was comparable in both groups (48.9% in iHTN_incr_, 40.0% in iHTN_imm_; *p* = 0.332). The type of induced hypertension was not independently associated with the occurrence of DCI-related infarction in a logistic regression model (odds ratio 1.004; 95% confidence interval 0.329–3.443; *p* = 0.942). Similar rates of pressor-treatment-related complications were observed in both treatment groups. Favorable outcome was reached in 44 (43.1%) patients in the immediate vs. 10 (27.0%) patients in the incremental treatment group (*p* = 0.076). However, only Hunt and Hess grading was identified as an independent predictor variable of clinical outcome (odds ratio 0.422; 95% confidence interval 0.216–0.824; *p* = 0.012).

**Conclusions:**

Immediate induction of hypertension with higher pressure targets did not result in a lower rate of DCI-related infarctions but was not associated with a higher complication rate compared with an incremental approach. Future tailored blood pressure management based on patient- and time-point-specific needs will hopefully better balance the neurological advantages versus the systemic complications of induced hypertension.

**Supplementary Information:**

The online version contains supplementary material available at 10.1007/s12028-022-01466-7.

## Introduction

Aneurysmal subarachnoid hemorrhage (SAH) is a devastating type of hemorrhagic stroke that can cause death or severe disability [1]. Despite being relatively rare, it affects young patients, and thereby the loss of productive years is comparable to more prevalent ischemic stroke [2, 3]. Apart from the severity of the initial hemorrhage, clinical outcome is mainly determined by the occurrence of delayed cerebral ischemia (DCI) and the success of treatment thereof [4].

DCI constitutes a complex multifactorial process of which angiographic narrowing of larger cerebral arteries is only one of the contributing factors. The acute increase in intracranial pressure after aneurysm rupture and short-lasting global cerebral hypoperfusion set a deleterious process in motion coined early brain injury [5]. Changes on a microvascular level due to endothelial injury with microspasm, blood–brain barrier disruption, and dysfunctional autoregulation all predispose the brain to further ischemic injury [6, 7]. If DCI is diagnosed early, treatment may prevent development of cerebral infarction and improve functional outcome [8, 9].

Historically recommended triple-H treatment has been abandoned in favor of isolated euvolemic hypertension (iHTN) [10]. Evidence of induced hypertension is based on clinical observation and observational cohort studies. Grounded in this level B evidence, most guidelines recommend the induction of hypertension to treat symptomatic DCI [11–13]. Artificial blood pressure increase causes straining of the entire cardiovascular system, and peripheral vasoconstriction can cause ischemic damage to liver, kidneys, intestine, and skin. Reported complications of induced hypertension range from a higher risk of myocardial infarction and congestive heart failure to hemorrhagic transformation of cerebral infarction and reversible leukoencephalopathy [14, 15]. The only existing randomized controlled trial comparing induced hypertension versus normotensive treatment was halted prematurely because of slow recruitment, lack of treatment effect, and the observed complication rate [16–18], Nonetheless, a retrospective analysis by Haegens et al. of 300 patients with SAH, of whom 201 were treated with induced hypertension, revealed a lower rate of DCI-related infarction compared with patients who were denied such treatment [19].

Official guidelines do not specify how to apply induced hypertension, which vasopressors to use, what target blood pressures to achieve, how to follow up treatment effects, and how to define patients refractory to iHTN. This is reflected by the heterogeneity of international treatment protocols [20]. The goal of this observational study was to compare two different iHTN treatment approaches: gradual titration of systolic blood pressure according to clinical demand versus immediate elevation of systolic blood pressure above 180 mm Hg for all patients with symptomatic DCI. It was anticipated that an immediate, more aggressive approach might be more effective in prevention of DCI-related infarction but might potentially cause a higher rate of complications.

## Methods

### Patient Population

This study constitutes an observational cohort study, for which data prior to 2014 were collected retrospectively, and entails a subgroup analysis of data partially published previously [8, 9]. Patients in this cohort have been analyzed before for the introduction of invasive neuromonitoring as a DCI detection tool and its effects on treatment and outcome after SAH. This study was designed as a two-group cohort analysis comparing two hypertensive treatment strategies for DCI. The data collection process was part of a previously registered study (NCT02142166) and was approved by the ethics committee of the Medical Faculty of the RWTH Aachen University (EK 062/14). All consecutive SAH cases presented in a single university hospital between 2010 and 2018 were considered for inclusion. Inclusion required verification of the aneurysmal cause by either computed tomography (CT) angiography or conventional cerebral angiography. Patients between 18 and 90 years of age were included. Patients were excluded in cases of cerebral infarction prior to iHTN induction, initial moribund presentation (mydriatic pupil for > 45 min or other signs of brain stem herniation), or global cerebral ischemia, all indicative of anticipated early mortality.

### Standard Treatment

Aneurysms were secured within 48 h via either surgical clipping or endovascular occlusion (coiling, flow-diverter stenting, or Woven EndoBridge (WEB) device placement) after diagnosis of hemorrhage. Patients were transferred to a dedicated neurointensive care unit for further observation. All patients were treated with prophylactic oral nimodipine and received an initial wake-up test for clinical DCI surveillance. If patients were clinically assessable, treatment of DCI was triggered whenever criteria of clinical DCI were fulfilled (new focal neurologic deficit or a decrease in the Glasgow coma scale ≥ 2 for at least 1 h, not ascribable to other diagnoses) [4]. If patients remained clinically not assessable, further DCI surveillance was performed by either repeated perfusion CT imaging or placement of invasive neuromonitoring with brain tissue oxygen monitoring (Neurovent PTO, Raumedic, Helmbrechts, Germany), cerebral microdialysis (71 High Cut-Off Brain Microdialysis Catheter, µdialysis, Stockholm, Sweden), or both. In perfusion CT imaging, DCI was defined as a perfusion deficit with a typical territorial cerebral blood flow/mean transit time (CBF/MTT) mismatch. In patients with invasive neuromonitoring, the definition of DCI was extended to include metabolic derangements (lactate/pyruvate ratio ≥ 40) or oxygenation crises brain tissue oxygen pressure (*p*_ti_O_2_ < 10 mm Hg or *p*_ti_O_2_ < 15 mm Hg in combination with lactate/pyruvate ratio ≥ 40) as described in our previous work [8, 9]. The cutoff of 10 mm Hg was chosen low to prevent overtreatment. Deflections above or below absolute predefined invasive monitoring cutoffs were always considered together with the slope of changes over time and treatment decisions were based on congruence between monitoring modalities.

On diagnosing DCI, first tier treatment consisted of induced euvolemic arterial hypertension by means of intravenous norepinephrine. At this point, the type of hypertensive treatment (incremental versus immediate elevation, see below) was chosen by the treating physician. All patients were equipped with a central venous access for norepinephrine infusion, and blood pressure was monitored continuously via an arterial line. Blood pressure data were recorded by means of a Philips IntelliVue MP70 Patient Monitor unit (Philips Medizin Systeme, Böblingen, Germany) and saved automatically in 5-min intervals into the IntelliSpace Critical Care and Anesthesia software. For further statistical purposes, pressures were averaged over 1-h intervals. Patient without clinical or radiological improvement during hypertensive treatment, persisting brain hypoxia, or anaerobic metabolism as measured in invasive monitoring were considered for endovascular rescue treatment. Last tier endovascular DCI treatment included balloon angioplasty in case of localized proximal vasospasm or spasmolysis with intraarterial nimodipine for diffuse distal angiographic vessel narrowing. The time of DCI onset along the time point of beginning induced hypertension was extracted from the electronic patient record.

### Induced Hypertension

Two hypertensive treatment strategies to reach blood pressure targets were applied during the inclusion time frame. In a subset of patients, systolic blood pressure goals were raised in 20-mm Hg increments beginning at 20 mm Hg above baseline systolic blood pressure on DCI diagnosis. In this incremental treatment group (iHTN_incr_), treatment effect was evaluated whenever stable elevated pressures were achieved. In case no improvement of clinical, radiological, or invasively detected DCI was observed, the systolic pressure goal was increased 20 mm Hg. This process was repeated until a systolic pressure above 180 mm Hg was considered necessary. In the remainder of patients, systolic blood pressure was immediately raised to reach systolic levels above 180 mm Hg (iHTN_imm_). In both treatment groups, further pressure augmentation above 200 mm Hg was considered if DCI symptoms persisted under systolic pressures over 180 mm Hg but only in absences of iHTN-induced complications and as long as pressure targets were tolerated by the cardiovascular system. The type of hypertensive treatment was chosen as per preference of the treating team, with a gradual shift away from incremental elevation over time during the inclusion time frame. Induced hypertension was continued until a clinical stable condition was reached. Weaning of induced hypertension was attempted when the DCI phase was deemed over. If symptoms reoccurred during weaning, norepinephrine was restarted and the reduction of its infusion rate was reattempted 24 h later. The total duration of iHTN treatment was noted as the number of days from treatment initiation until complete weaning.

### Outcome Definition

The occurrence of DCI-related infarction was chosen as the primary outcome and was defined as a new region of hypodensity on CT imaging after DCI onset not attributable to any other cause (e.g., aneurysm treatment complication, embolic origin). This primary outcome was assessed by two independent assessors (MV and MW) blinded to treatment allocation, and discrepancies in findings were discussed until consensus was reached. We anticipated finding a higher rate of DCI-related infarction in the iHTN_inc_ group.

The need for endovascular rescue treatment, as well as long-term clinical outcome, was chosen as a secondary outcome. Long-term clinical outcome was measure by the extended Glasgow outcome scale (GOS-E) after 12 months and was dichotomized into unfavorable (GOS-E_1-4_) and favorable (GOS-E_5-8_) outcome. A blinded assessor prospectively collected clinical outcome data in a structured telephone interview. In patients included prior to 2014, outcome data were collected during regular follow-ups and missing information was appended by analysis of patient files. Because of the anticipated higher infarction rate in the iHTN_incr_ group, a higher rate of patients with unfavorable outcome was expected. Potential complications of hypertensive treatment were noted and included pulmonary edema, congestive heart failure, intestinal ischemia, and posterior reversible encephalopathy (PRES).

### Statistical Analysis

 Data are presented as mean and standard deviation for normally distributed continuous variables and as median and interquartile range for nonnormally distributed continuous variables. Categorical variables are depicted as frequencies and proportions. After normality testing via plotting and the Shapiro–Wilk test, the appropriate statistical test was selected. For nominal data, the *χ*^2^ test was used; for normally distributed continuous data, the independent sample *t*-test was used; and for nonnormally distributed data, the Mann–Whitney *U*-test was chosen. The effects of treatment groups on the development of DCI-related infarction, need for rescue treatment, and occurrence of favorable outcome were assessed in a logistic regression model. Predictor covariates were included in case of univariate results presented with a *p* value < 0.1 Prior to inclusion, numeric data were tested for multicollinearity via the Box-Tidwell procedure and potential outliers were identified. For the analysis of continuous variables between groups measured over multiple time points, a two-way repeated measures analysis of variance was run with adjustment via the Greenhouse-Geissner correction. Missing outcome data were not imputed. All statistical analyses were performed using IBM SPSS Statistics 25 (SPSS Inc., Chicago, IL), and graphics were plotted using GraphPad Prism 9.0.1 (GraphPad Software, Inc., La Jolla, CA). Statistical significance was defined as a two-sided *p* value < 0.05.

### Sources of Bias

The main sources of anticipated bias are the lack of randomization and selection bias, which is introduced by the per-preference treatment allocation. To address selection bias, both treatment groups will be compared for all outcomes (DCI infarction rate and GOS-E) and relevant baseline and disease-specific characteristics, e.g., age, Hunt and Hess grading, modified Fisher grading, aneurysm location, aneurysm occlusion modality, etc. Because of the long inclusion time frame (8 years), performance bias, especially in relation to DCI treatment and last tier endovascular therapy, is expected. Both treatment groups will be compared regarding the need for and the modality of endovascular rescue treatment. Potential reporting bias, especially in the retrospective collected data (inclusion prior to 2014) might be introduced because of incomplete or ambiguous data recording. This can only be addressed by rigorous analysis of patients’ files but mainly depends on the quality their off.

## Results

### Patients

**Fig. 1:**
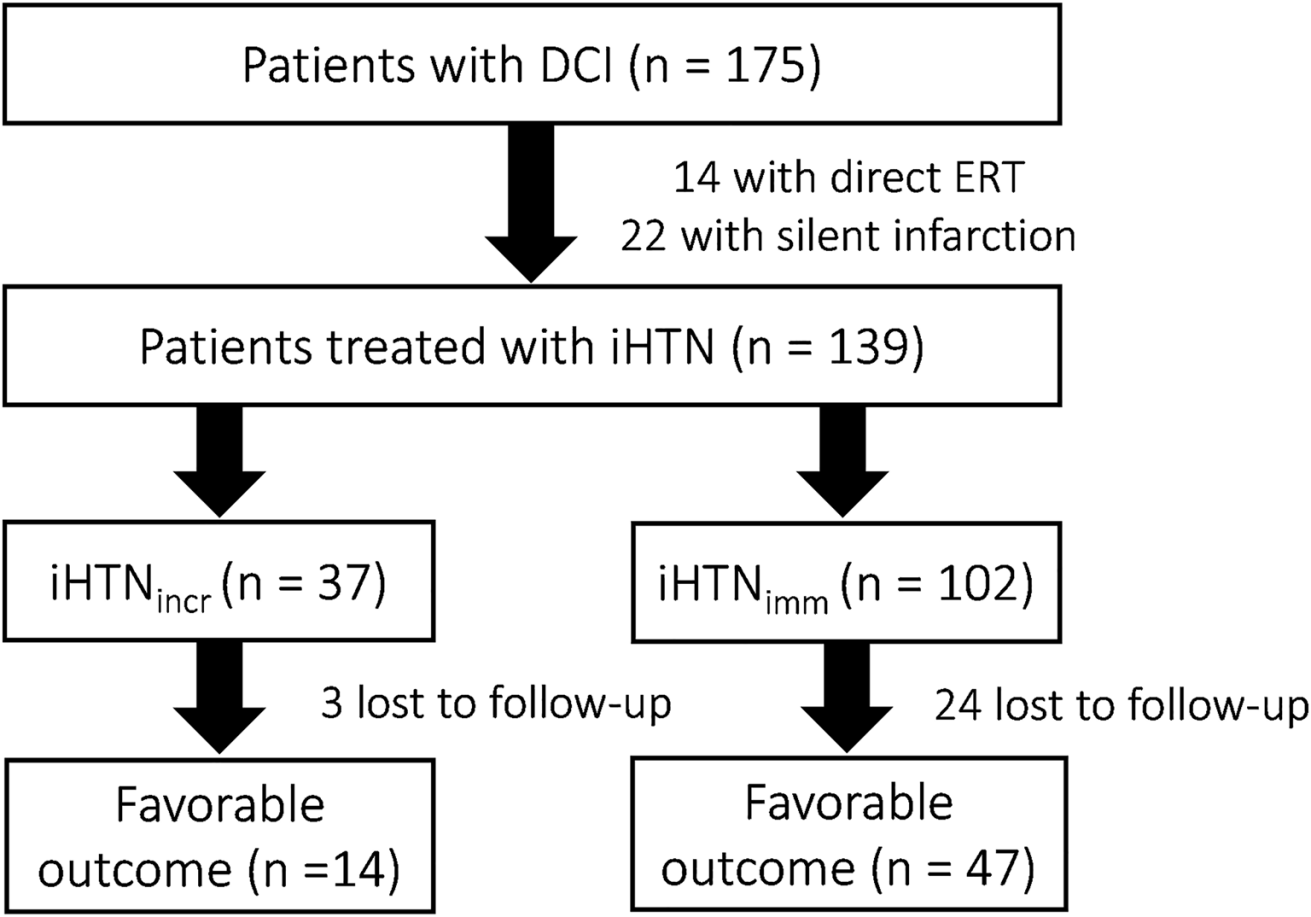
Inclusion flowchart. DCI, delayed cerebral ischemia; ERT, endovascular rescue treatment; iHTN_imm_, immediate induced hypertension treatment group; iHTN_incr_, incremetal induced hypertension treatment group

Of all 325 analyzed consecutive patients with SAH, 175 (53.8%) were diagnosed with DCI according to our modified definition (including patients with aberrant deflection in invasively measured parameters, i.e., *p*_ti_O_2_ and lactate/pyruvate ratio). Fourteen patients did not receive hypertensive treatment because the severity of angiographic vasospasm prompted direct endovascular balloon angioplasty or intraarterial spasmolysis. An additional 22 patients were excluded because the first sign of ongoing DCI was an already demarcated cerebral infarction. A total of 139 patients were treated with iHTN and included in the final analysis. An overview of patients’ inclusion is provided in a flowchart (Fig. 1).

 In 37 patients (26.6%), systolic blood pressure was elevated in 20-mm Hg increments (iHTN_incr_) with reevaluation of treatment effect (clinically via either perfusion CT imaging or invasive neuromonitoring) after achieving a stable augmented blood pressure. In the remainder of patients (*n* = 102; 73.4%), blood pressure was immediately elevated to reach systolic values above 180 mm Hg (iHTN_imm_).

The mean age of included patients was 53.9 ± 12.0 years, of whom 100 (71.9%) were female and 39 (28.1%) were male. Hemorrhage severity according to Hunt and Hess grading as well as the modified Fisher scale proved comparable between both treatment groups. Additional relevant patient- and disease-specific baseline data are presented in Table 1. No significant differences in baseline characteristics were observed between treatment groups.

**Table 1:** Overview and comparison between treatment groups of patient- and hematoma-specific baseline data

	All iHTN (*n* = 139)	Incremental iHTN (*n* = 37)	Immediate iHTN (*n* = 102)	*p*- value
Demographics				
Age-yrs.-mean ± SD (range)	53.9 ± 12.0	51.5 ± 11.5	54.8 ± 12.1	0.148
Sex-female/male	100 (71.9)/39 (28.1)	27 (73.0)/10 (27.0)	73 (71.6)/29 (28.4)	0.871
Prior hypertension	54 (38.8)	10 (27.0)	44 (43.1)	0.085
Aneurysm location-no. (%)				
Ant. circulation	118 (84.9)	32 (86.5)	86 (84.3)	0.787
Post. circulation	21 (15.1)	5 (13.5)	16 (15.8)	
Multiple aneurysms	42 (30.2)	16 (43.2)	26 (25.5)	0.044
Aneurysm max. diameter (mm)	7.0 ± 3.3	7.2 ± 3.0	7.0 ± 3.5	0.710
Hemorrhage severity				
Hunt and Hess grade-no. (%)				0.091
Grade 1	14 (10.1)	3 (8.1)	11 (10.8)	
Grade 2	29 (20.9)	3 (8.1)	26 (25.5)	
Grade 3	49 (35.3)	13 (35.1)	36 (35.3)	
Grade 4	31 (22.3)	13 (35.1)	18 (17.6)	
Grade 5	16 (11.5)	5 (13.5)	11 (10.8)	
Modified Fisher scale-no. (%)				0.285
Grade 1	20 (14.4)	2 (5.4)	18 (17.6)	
Grade 2	18 (12.9)	5 (13.5)	13 (12.7)	
Grade 3	42 (30.2)	11 (29.7)	31 (30.4)	
Grade 4	59 (42.4)	19 (51.4)	40 (39.2)	
Aneurysm occlusion-no. (%)				
Clipping/endovascular	68 (48.9)/71 (51.1)	21 (56.8)/16 (43.2)	47 ( 46.1)/55 (53.9)	0.266
DCI surveillance				
INM-no. (%)	71 (51.1)	13 (35.1)	58 (56.9)	0.698
ptiO_2_	70 (50.4)	13 (35.1)	57 (55.9)	0.375
CMD	57 (41.0)	8 (21.6)	49 (48.0)	0.436

CMD, cerebral microdialysis; DCI, delayed cerebral ischemia; iHTN, induced hypertension; INM, invasive neuromonitoring; ptiO_2_, brain tissue oxygen tension; SD, standard deviation

### DCI Treatment

The total duration of iHTN was comparable in both treatment groups (iHTN_incr_ 10.1 ± 6.7 days vs. iHTN_imm_ 9.6 ± 6.3 days; *p* = 0.703). Fourteen (37.8%) patients remained refractory to hypertensive treatment and received endovascular rescue therapy for DCI in the incremental treatment group versus 55 (53.9%) patients in the iHTN_imm_ treatment group (*p* = 0.094). A significantly higher number of patients in the immediate group were treated with continuous intraarterial nimodipine (iHTN_incr_ 3 [8.1%] vs. iHTN_imm_ 24 [23.5%]; *p* = 0.042) (Table 2).

**Table 2:** Comparison of treatment results between incremental and immediate induced hypertension

	Incremental iHTN (*n* = 37)	Immediate iHTN (*n* = 102)	*p*-value
Initial DCI detection-no. (%)			0.034
Clinical DCI	11 (29.7)	51 (50.0)	
Technical DCI	27 (70.3)	51 (50.0)	**< 0.001**
Duration iHTN-days	10.1 ± 6.7	9.6 ± 6.3	0.703
iHTN complications-no. (%)			
Pulmonary Eedema	12 (32.4)	20 (19.6)	0.112
Congestive heart failure	4 (10.8)	12 (11.8)	0.876
Intestinal Ischemia	0 (0)	1 (1.0)	
PRES	2 (5.4)	1 (1.0)	
DCI refractory to iHTN			
ERT	14 (37.8)	55 (53.9)	0.094
Spasmolysis	11 (29.7)	43 (42.2)	0.184
Angioplasty	4 (10.8)	10 (9.8)	0.862
Continuous intra-arterial nimodipine	3 (8.1)	24 (23.5)	**0.042**
Clinical outcome-no. (%)			
GOS-E 12 months			0.227
Upper good recovery	1 (2.7)	6 (5.9)	
Lower good recovery	1 (2.7)	5 (4.9)	
Upper moderate disability	3 (8.1)	16 (15.7)	
Lower moderate disability	5 (13.5)	17 (16.7)	
Upper sever disability	2 (5.4)	8 (7.8)	
Lower sever disability	8 (21.6)	8 (7.8)	
Vegetative state	4 (10.8)	5 (4.9)	
Dead	11 (29.7)	20 (19.6)	
Favorable outcome	10 (27.0)	44 (43.1)	0.076
Unfavorable outcome	25 (54.1)	41 (40.2)	
Missing outcome data	2 (5.4)	17 (16.7)	
DCI related infarction	15 (40.5)	21 (20.6)	**0.046**
DCI related mortality	2 (5.4)	3 (2.9)	0.775

Results reaching statistical significance (*p*-value < 0.05) are written in bold

DCI, delayed cerebral ischemia; ERT, endovascular rescue treatment; GOS-E, extended Glasgow outcome scale; iHTN, induced hypertension; PRES, posterior reversible encephalopathy syndrome

### Temporal Course of Blood Pressure

**Fig. 2:**
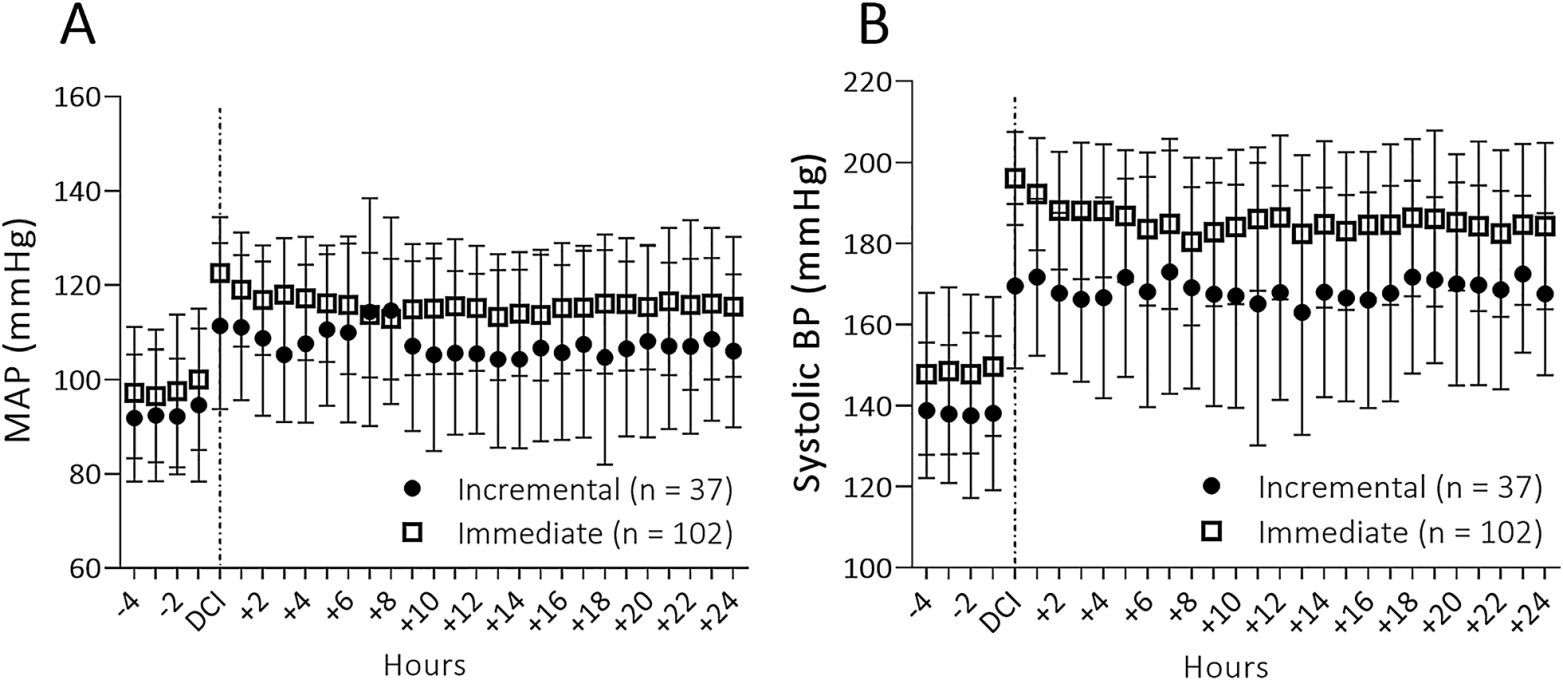
Mean arterial and systolic pressure over time (hours) in both iHTN treatment groups. **a** Systolic blood pressure (mean ± SD) synchronized around the initial DCI event triggering hypertensive treatment (hours). **b** Mean arterial blood pressure (mean ± SD) synchronized around the initial DCI event triggering hypertensive treatment (hours). BP, blood pressure; DCI, delayed cerebral ischemia; MAP, mean arterial pressure; SD, standard deviation

When plotting hourly blood pressures over time it becomes clear that the differences in treatment manifest themselves early after DCI is diagnosed. A trend toward a significant difference was seen in hourly systolic pressures when we compared the two approaches (*F*_28,3457_ = 1.458; *p* = 0.057). Mean hourly arterial pressures were significantly higher over time during the first 24 h in the iHTN_imm_ group (*F*_28,3435_ = 1.903; *p* = 0.003) (Fig. 2a, b).

**Fig. 3:**
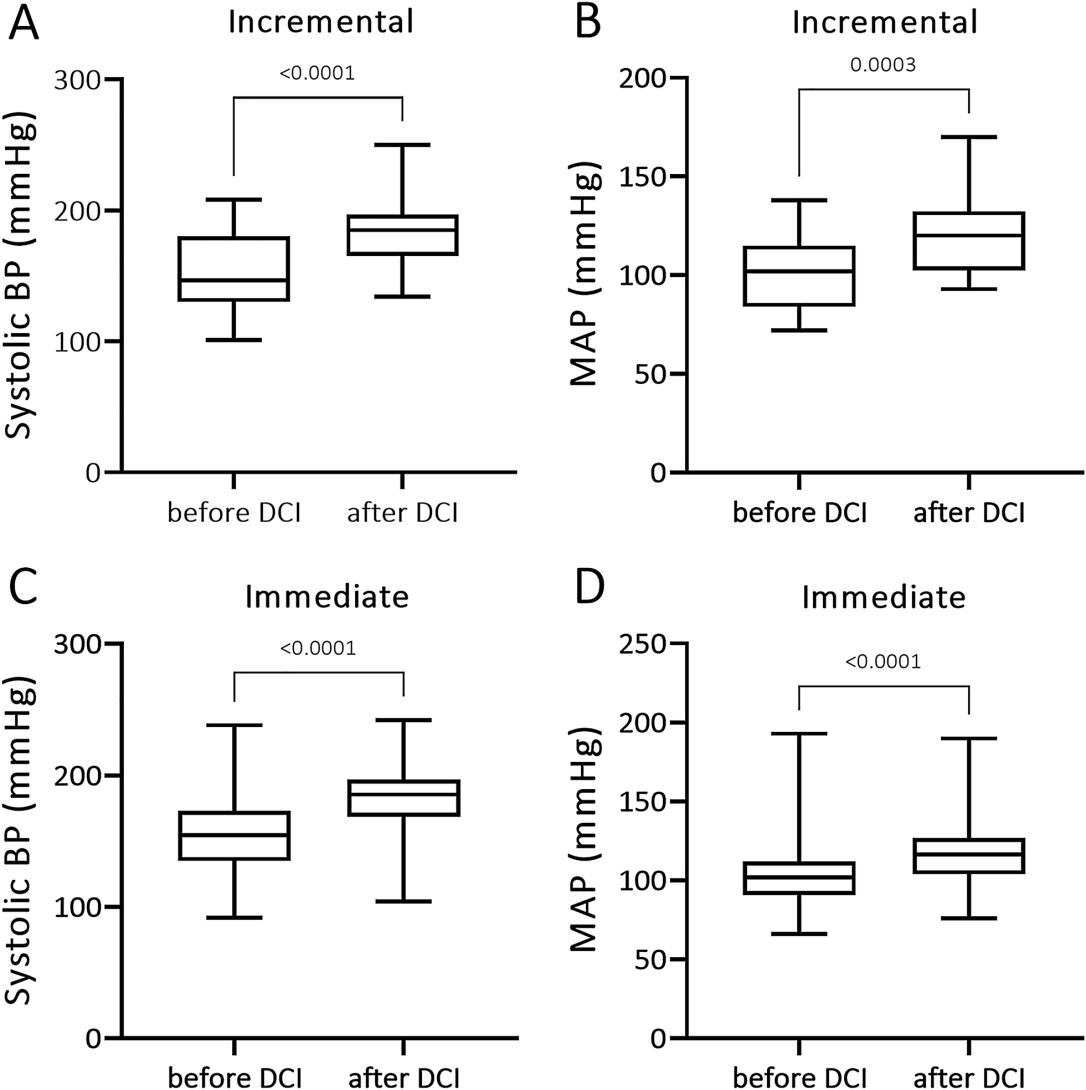
Box plot (median, interquartile range, and range) comparison of blood pressure 24 h before DCI onset and 24 h after beginning of treatment with induced hypertension. **a** Systolic blood pressure before (24 h) and after (24 h) DCI treatment with incremental induced hypertension. **b** Mean arterial pressure before (24 h) and after (24 h) DCI treatment with incremental induced hypertension. **c** Systolic blood pressure before (24 h) and after (24 h) DCI treatment with immediate induced hypertension. **d** Mean arterial pressure before (24 h) and after (24 h) DCI treatment with immediate induced hypertension. BP blood pressure; DCI, delayed cerebral ischemia

In a two-group comparison of pressures 24 h before DCI diagnosis and 24 h after treatment initiation, a significant increase of both systolic and mean arterial pressure was observed in both the incremental and immediate groups (Fig. 3a–d).

### Complications of Hypertensive Treatment

Prevalence of complications associated with hypertensive treatment was compared between both treatment groups. Rates of pulmonary edema (iHTN_incr_ 32.4% vs. iHTN_imm_ 19.6%; *p* = 0.112) and congestive heart failure (10.8% vs. 11.8%; *p* = 0.876) were comparable. A single case of intestinal ischemia was observed in the iHTN_imm_ group. PRES was seen in two cases of the iHTN_incr_ group and in a single case in the iHTN_imm_ group. Increased intracranial pressure was registered in 10 (27.0%) patients in the iHTN_incr_ and in 27 (26.5%) patients in the iHTN_imm_ group (*p* = 0.948) (Table 2). Onset of increased intracranial pressure preceded the need for hypertensive treatment in most cases and only occurred during iHTN in 1 (2.7%) patient in the iHTN_imm_ group and in 2 (2.0%) patients in the iHTN_incr_ group (*p* = 0.790). Intracranial hypertension during hypertensive treatment as an isolated symptom did not result in the adjustment of hypertensive treatment strategy in this cohort.

### Refractory DCI and Endovascular Rescue Treatment

Univariate testing identified sex (*p* = 0.080), presence of invasive neuromonitoring (*p* = 0.004), Hunt and Hess grading (*p* = 0.059), and the type of applied iHTN treatment (iHTN_incr_ vs. iHTN_imm_) (*p* = 0.094) as covariates to be introduced into the logistic regression model. Of these predictor variables, presence of invasive neuromonitoring and the Hunt and Hess grade cover overlapping information because mainly high-grade patients were equipped with invasive neuromonitoring. Therefore only Hunt and Hess grading was introduced into the prediction model. This resulted in a model that was not statistically significant $\chi_{3}^{2} = 7.051$, *p* = 0.070; precluding further interpretation of results regarding the predictor covariates (Supplementary Table S1).

### DCI-Related Infarction

Univariate testing identified age (*p* = 0.037), presence of invasive neuromonitoring (*p* = 0.072), and iHTN treatment group (*p* = 0.035) as covariates to be introduced into the logistic regression model assessing DCI-related infarction (Table 3). Of the included predictor variables, only age was a significantly associated with DCI-related infarction (odds ratio 1.043; 95% confidence interval 1.003–1.083; *p* = 0.035) (Supplementary Table S1) (Table 4).

**Table 3:** Univariate comparison of patient with or without the development of DCI-related infarction

	Nno DCI- related infarction (*n* = 101)	DCI- related infarction (*n* = 38)	*p*- value
Demographics			
Age-yrs.-mean ± SD (range)	52.6 ± 12.4	57.4 ± 10.5	**0.037**
Sex-female/male	73 (72.3)/28 (27.7)	27 (71.1)/11 (28.9)	0.886
Aneurysm location-no. (%)			
Ant. circulation	84 (83.2)	34 (89.5)	0.355
Post. Circulation	17 (16.8)	4 (10.5)	
Aneurysm occlusion-no. (%)			
Clipping/endovascular	49 (48.5)/52 (51.5)	19 (50.0)/19 (50.0)	0.876
DCI surveillance			
INM-no. (%)	49 (48.5)	22 (57.9)	0.072
Hemorrhage severity			
Hunt and Hess grade-no. (%)			0.250
Grade 1	11 (10.9)	3 (7.9)	
Grade 2	24 (23.8)	5 (13.2)	
Grade 3	37 (36.6)	12 (31.6)	
Grade 4	18 (17.8)	13 (34.2)	
Grade 5	11 (10.9)	5 (13.2)	
Modified Fisher scale-no. (%)			0.164
Grade 1	18 (17.8)	2 (5.3)	
Grade 2	11 (10.9)	7 (18.4)	
Grade 3	28 (27.7)	14 (36.8)	
Grade 4	44 (43.6)	15 (39.5)	
iHTN treatment group-no. (%)			**0.035**
Incremental iHTN (n = 37)	22 (21.8)	15 (39.5)	
Immediate iHTN (n = 102)	79 (78.2)	23 (60.5)	

Results reaching statistical significance (*p*-value < 0.05) are written in bold

DCI, delayed cerebral ischemia; iHTN, induced hypertension; INM, invasive neuromonitoring

### Clinical Outcome

**Fig. 4:**
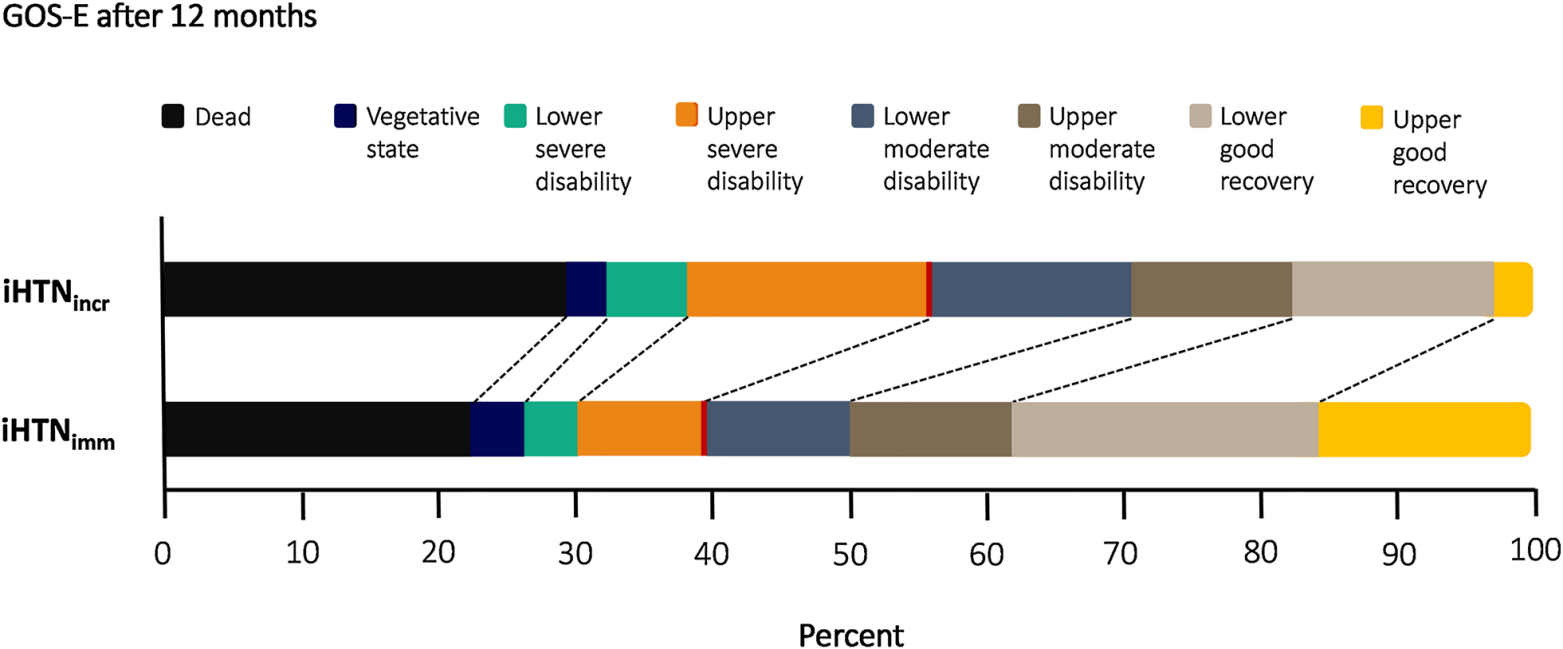
The GOS-E in both induced hypertension treatment groups after 12 months. GOS-E, extended Glasgow outcome scale; iHTN_imm_, immediate induced hypertension treatment group; iHTN_incr_, incremetal induced hypertension treatment group

Long-term clinical outcome as measured via the GOS-E after 12 months, was missing for 2 (5.4%) patients in the incremental treatment group and for 17 (16.7%) patients in the immediate treatment groups. Favorable outcome was reached in 44 (43.1%) patients in the immediate treatment group vs. 10 (27.0%) patients in the incremental treatment group (*p* = 0.076) (Fig. 4). Univariate testing identified aneurysm location (*p* = 0.068), presence of invasive neuromonitoring (*p* < 0.001), Hunt and Hess grading (*p* < 0.001), the modified Fisher scale (*p* < 0.001), and iHTN treatment group (*p* = 0.076) as covariates to be introduced into the logistic regression model assessing dichotomized clinical outcome after 12 months (GOS-E) (Table 4). Only Hunt and Hess grading was identified as an independent predictor variable of clinical outcome (odds ratio 0.422; 95% confidence interval 0.216–0.824; *p* = 0.012) (Supplementary Table S1).

**Table 4:** Univariate comparison of patient with unfavorable or favorable outcome, as measured via the extended Glasgow outcome scale after 12 months

	Unfavorable outcome (*n* = 53)	Favorable outcome (*n* = 62)	*p*- value
Demographics			
Age-yr-mean ± SD (range)	54.9 ± 12.2	52.2 ± 11.5	0.231
Sex-female/male	38 (71.7) / 15 (28.3)	45 (72.6) / 17 (27.4)	0.916
Aneurysm location-no. (%)			0.068
Ant. circulation	49 (92.5)	50 (80.6)	
Post. circulation	4 (7.5)	12 (19.4)	
Aneurysm occlusion-no. (%)			
Clipping/endovascular	25 (47.2) / 28 (52.8)	30 (48.4) / 32 (51.6)	0.896
DCI surveillance			
INM-no. (%)	31 (58.5)	26 (41.9)	< 0.001
Hemorrhage severity			
Hunt and Hess grade-no. (%)			< 0.001
Grade 1	1 (1.9)	8 (12.9)	
Grade 2	1 (1.9)	20 (32.3)	
Grade 3	21 (39.6)	23 (37.1)	
Grade 4	20 (37.7)	8 (12.9)	
Grade 5	10 (18.9)	3 (4.8)	
Modified Fisher scale-no. (%)			< 0.001
Grade 1	1 (1.9)	17 (27.4)	
Grade 2	4 (7.5)	11 (17.7)	
Grade 3	19 (35.8)	12 (19.4)	
Grade 4	29 (54.7)	22 (35.5)	
iHTN treatment group-no. (%)			0.076
Incremental iHTN (n = 37)	20 (37.7)	14 (22.6)	
Immediate iHTN (n = 102)	33 (62.3)	48 (77.4)	

DCI, delayed cerebral ischemia; ERT, endovascular rescue therapy; iHTN, induced hypertension; INM, invasive neuromonitoring

## Discussion

We investigated the effects of two different approaches for induction of hypertension once DCI was diagnosed in patients with aneurysmal SAH. The goal was to quantify systematic differences in efficacy and complication rate with immediate induction of hypertension rather than with an incremental increase. It was anticipated that slower up-titration of hypertensive treatment (as in the iHTN_incr_ treatment group) would results in a slower achievement of adequate perfusion pressure and longer lasting misery perfusion. Hereby, ischemic processes are potentially allowed to develop further, leading to higher rate of DCI-related infarctions. The type of induction in this study was not independently associated with the occurrence of DCI-related infraction, neither the rate of favorable outcome nor the need for endovascular rescue treatment. Older age was identified as a predictor of DCI-related infarction and higher Hunt and Hess grading as the only independent predictor of unfavorable long-term outcome. Nevertheless, immediate induction of hypertension was not associated with a higher rate of complications typical for induced hypertension.

Blood pressure remained generally lower in the incremental group because further elevation of pressure targets was not considered necessary, as an improvement of symptoms was observed. Although hypertensive treatment targets were based on systolic pressure, only a nonsignificant difference in mean arterial pressure was identified between both treatment approaches when we compared blood pressures over time. Differences in measured pressures between treatment groups were apparent early after treatment initiation (hours) and evened out later (days) as pressure targets were progressively increased in the incremental group. Both treatment strategies constituted a gradual change with overlap in time of our institutional treatment algorithm. Because anecdotal observations were indicative of better DCI treatment efficacy of immediate pressure elevation, there was a shift away from incremental blood pressure increase, and the systolic blood pressure target > 180 mm Hg has been fixated in our institutional protocol.

There exist two major confounders contributing to significant performance bias in the presented data. We already demonstrated a reduction of DCI-related infarction in our SAH cohort after introduction of invasive neuromonitoring into our DCI diagnostic algorithm [8, 9]. Although the proportions of patients equipped with invasive monitoring were statistically not different between the incremental and immediate groups. Also, there was a higher rate of patients treated with continuous intraarterial nimodipine in the immediate elevation group. A relevant effect of this procedure by reducing infarction rates and improving outcome is anticipated.

In the first randomized controlled trial comparing induced hypertension with normotensive pressure management for DCI treatment, cerebral blood flow was measured in regular time intervals via perfusion CT imaging [17]. Because no significant changes in overall cerebral blood flow were observed, the authors concluded that induction of hypertension should not be recommended to augment overall cerebral blood flow in patients with SAH suffering from DCI. The HIMALAIA trial was, however, halted prematurely because of these results along slow recruitment with the inclusion of 41 patients over 6 years of time. Poor outcome occurred in 57% of patients in the hypertension group and in 40% of patients in the group without hypertension [18]. In the HIMALAIA trial, pressure goals were a mean arterial pressure exceeding 120 mm Hg or a systolic pressure above 230 mm Hg. These treatment goals well exceeded the target blood pressures set to achieve in our study. The high rate of severe adverse events reported in the HIMALAIA trial also contributed to its premature termination. However, severe adverse events were broadly defined and were mostly nonspecific for iHTN treatment (e.g., pneumothorax, pneumosepsis and death). The long intensive care unit stay of patients with SAH alone is a major contributor to complications, as well as the often dramatic course DCI may take independent of treatment. Additionally, the more aggressive nature of the applied iHTN could have contributed to specific complications, such as pulmonary edema, heart failure, intracranial pressure (ICP) crises, peripheral ischemia, and PRES, that were not specifically reported.

In contrast to the results above, a retrospective analysis by the same group of authors of 201 patients treated with hypertensive treatment and 99 patients without identified a lower infarction rate in patients treated with incremental increase of blood pressure (20%) compared with 33% in patients without induced hypertension. In this large cohort, however, only the amount of subarachnoid blood was identified as an independent predictor of the occurrence of DCI-related cerebral infarction and not hypertensive treatment.

We anticipated a higher rate of complications in our cohort of patients with immediate pressure elevation. This was, however, not confirmed, possibly because of the fact that blood pressure management was similar once a stable clinical state was reached. Other intensive-care-unit-specific outcome prediction factors, such as the duration of ventilation, development of nosocomial infections, and septic shock, possibly overshadow the effects minor differences in blood pressure management have on the development of systemic complications. PRES as a rare but specific complication of induced hypertension was observed in both treatment groups [15]. Also, the long-term effects of hypertensive treatment due to excessive straining of the cardiovascular system are still unknown.

Despite its observed effectiveness, the exact pathophysiological mechanisms that underlie cerebral blood flow increase by hypertensive treatment in patients with SAH is incompletely understood. Under normal physiological conditions, cerebral autoregulation would counteract increased blood flow by cerebral arteriolar vasoconstriction. Most possibly, the concomitant loss of autoregulation due to early brain injury in SAH is one of the factors, which makes cerebral blood flow augmentation possible.

Apart from the obvious limitations inherent to the retrospective design of this study, a reporting bias could have been introduced, as side effects of treatment may be underreported or the causality toward induced hypertension may not be documented. Because the choice of treatment was up to the clinician’s preference, there might have been a preference for incremental treatment in patients with an already existing need for vasopressor treatment prior to DCI detection, introducing selection bias. This introduces a preference toward patients with already manifested systemic disease for this treatment group. Vice versa, immediate induced hypertension could have been chosen more frequently in patients with already manifesting permissive hypertension as a sign of ongoing DCI. Additionally, preexisting hypertension was not taken into consideration neither in the decision for which treatment to pursue nor in the analysis of data. These patients are accustomed to higher systolic blood pressures and may require direct higher pressure targets compared with patients without hypertension. Finally, this study compares two different approaches in setting pressure targets and not actual reached blood pressures. Considerable overlap in actual pressures between both treatment groups exists and is mainly the result of differences in baseline blood pressure in the incremental group and fluctuations over time despite rigorous pressure targets. Also, both treatment protocols target systolic blood pressure, and treatment protocols focusing on mean arterial pressure have been suggested. Systolic pressure was still preferred owing to a higher familiarity with absolute values. Nevertheless the results of this study resulted in a fixed application of immediate blood pressure elevation above 180 mm Hg on DCI diagnosis as part of our institutional treatment algorithm. These data can help to optimize an induced hypertension treatment protocol for a randomized trial applying more patient- and situation-tailored pressure targets to avoid adverse events of overtreatment.

On the basis of our data, no definitive recommendation can be made on which treatment approach is best. However, setting initial higher pressure targets did not result in a higher rate of systemic or cerebral complications in this comparative analysis.

## Conclusions

Immediate induction of hypertension with higher pressure targets did not result in a lower rate of DCI-related infarctions and was not associated with a higher complication rate compared with an incremental approach. Future tailored blood pressure management based on patient- and time-point-specific needs will hopefully better balance the neurological advantages versus the systemic complications of induced hypertension for treatment of DCI.

## Supplementary Information

Below is the link to the electronic supplementary material.

Supplementary file 1 (DOCX 17 kb)

## Data Availability

The raw data on which this analysis is based can be made available by the authors to any qualified researcher on reasonable request.

## Abbreviations

CT: Computed tomography
DCI: Delayed cerebral ischemia
GOS-E: Extended Glasgow outcome scale
iHTN: Euvolemic induced hypertension
PRES: Posterior reversible encephalopathy syndrome
SAH: Aneurysmal subarachnoid hemorrhage

## Clinical trial registration

The prospective data collection was registered on May 20, 2014, at ClinicalTrials.gov (NCT02142166) as part of a larger scale prospective data collection.
