# Supplementary material for: Incremental Versus Immediate Induction of Hypertension in the Treatment of Delayed Cerebral Ischemia After Subarachnoid Hemorrhage
Source: Neurocrit Care. 2022 Mar 8;36(3):702–14. doi: 10.1007/s12028-022-01466-7 (PMC9110507; doi:10.1007/s12028-022-01466-7)
Supplement: Supplementary file 1 — (DOCX 17 kb) [file 12028_2022_1466_MOESM1_ESM.docx]

**Supplemental Table 1.**

|  | **no ERT (n = 70)** | **ERT (n = 69)** | **p-value** |
| --- | --- | --- | --- |
| **Demographics** |  |  |  |
| Age - yr - mean ± SD (range) | 55.4 ± 13.5 | 52.4 ± 10.2 | 0.151 |
| Sex - Female / Male | 55 (78.6) / 45 (64.3) | 45 (65.2) / 24 (34.8) | 0.080 |
| **Aneurysm location - no. (%)** |  |  | 0.251 |
| Ant. circulation | 57 (81.4) | 61 (88.4) |  |
| Post. circulation | 13 (18.6) | 8 (11.6) |  |
| **Aneurysm occlusion - no. (%)** |  |  | 0.934 |
| Clipping / Endovascular | 34 (48.6) / 36 (51.4) | 34 (49.3) / 35 (50.7) |  |
| **DCI surveillance** |  |  |  |
| INM - no. (%) | 27 (38.6) | 44 (63.8) | 0.004 |
| **Hemorrhage severity** |  |  |  |
| **Hunt and Hess grade - no. (%)** |  |  | 0.059 |
| Grade 1 | 8 (11.4) | 6 (8.7) |  |
| Grade 2 | 14 (20.0) | 15 (21.7) |  |
| Grade 3 | 19 (27.1) | 30 (43.5) |  |
| Grade 4 | 16 (22.9) | 15 (21.7) |  |
| Grade 5 | 13 (18.6) | 3 (4.3) |  |
| **Modified Fisher scale - no. (%)** |  |  | 0.478 |
| Grade 1 | 8 (11.4) | 12 (17.4) |  |
| Grade 2 | 11 (15.7) | 7 (10.1) |  |
| Grade 3 | 19 (27.1) | 23 (33.3) |  |
| Grade 4 | 32 (45.7) | 27 (39.1) |  |
| **iHTN treatment group - no. (%)** |  |  | 0.094 |
| Incremental iHTN (n = 37) | 23 (32.9) | 14 (20.3) |  |
| Immediate iHTN (n = 102) | 47 (67.1) | 55 (79.7) |  |

**Supplemental Table 1.** Univariate comparison of patient with or without the need for endovascular rescue treatment due to DCI refractory to hypertensive treatment.

DCI, delayed cerebral ischemia; ERT, endovascular rescue therapy; iHTN, induced hypertension; INM, invasive neuromonitoring.

**Supplemental Table 2.**

|  | **no ERT (n = 70)** | **ERT (n = 69)** | **OR** | **95% CI** | **p-value** |
| --- | --- | --- | --- | --- | --- |
| Sex - Female / Male | 55 (78.6) / 45 (64.3) | 45 (65.2) / 24 (34.8) | 0.667 | 0.276 to 1.611 | 0.368 |
| INM | 27 (38.6) | 44 (63.8) | 0.221 | 0.087 to 0.559 | **0.001** |
| Hunt and Hess grading |  |  | 0.735 | 0.506 to 1.068 | 0.107 |
| iHTN treatment group |  |  | 0.553 | 0.192 to 1.587 | 0.270 |
|  | **no DCI related infarction (n = 101)** | **DCI related infarction (n = 38)** | **OR** | **95% CI** | **p-value** |
| Age - yr - mean ± SD (range) | 52.6 ± 12.4 | 57.4 ± 10.5 | 1.042 | 1.003 to 1.083 | **0.035** |
| INM - no. (%) | 49 (48.5) | 22 (57.9) | 0.405 | 0.145 to 1.127 | 0.084 |
| iHTN treatment group - no. (%) |  |  | 1.004 | 0.329 to 3.443 | 0.942 |
|  | **Unfavorable outcome (n = 53)** | **Favorable outcome (n = 62)** | **OR** | **95% CI** | **p-value** |
| Aneurysm location (AC/PC) - no. (%) | 49 (92.5) / 4 (7.5) | 50 (80.6) / 12 (19.4) | 4.679 | 0.638 to 34.323 | 0.129 |
| Clipping / Endovascular | 25 (47.2) / 28 (52.8) | 30 (48.4) / 32 (51.6) | 1.108 | 0.368 to 3.335 | 0.855 |
| INM - no. (%) | 31 (58.5) | 26 (41.9) | 4.893 | 1.267 to 18.895 | 0.021 |
| Hunt and Hess grade - no. (%) |  |  | 0.422 | 0.216 tp 0.824 | **0.012** |
| Modified Fisher scale - no. (%) |  |  | 0.666 | 0.383 to 1.160 | 0.152 |
| iHTN treatment group - no. (%) | 20 (37.7) / 33 (62.3) | 14 (22.6) / 48 (77.4) | 0.525 | 0.129 to 2.136 | 0.368 |

**Supplemental Table 2.** Analysis of the effects of multiple predictor covariates on the need for ERT, occurrence of DCI related infarction and dichotomized outcome in a logistic regression model.

CI, confidence interval; ERT, endovascular rescue therapy; iHTN, induced hypertension; INM, invasive neuromonitoring; OR, odds ratio.
